# Chylothorax due to thrombosis of the jugular and subclavian veins in a patient with gastric cancer: a case report

**DOI:** 10.1186/s13256-023-03775-7

**Published:** 2023-03-04

**Authors:** M. Ünal, E. Ünal, A. L. T. Imholz

**Affiliations:** 1 Department of Internal Medicine, University Medical Center Groningen UMCG, P.O. Box 30.001, 9713 GZ Groningen, The Netherlands; 2 Department of Internal Medicine, Hospital Group Twente, Almelo, The Netherlands; 3 Department of Internal Medicine, Deventer Hospital, Deventer, The Netherlands

**Keywords:** Chylothorax, Thrombosis, Gastric cancer

## Abstract

**Background:**

Chylothorax is a rare condition due to leakage of chyle in the thoracic cavity. When large amounts of chyle leak into the thoracic cavity, it can lead to severe respiratory, immune, and metabolic complications. Chylothorax has many potential underlying etiologies, and the most common causes are traumatic chylothorax and lymphoma. Venous thrombosis of the upper extremities is a rare cause of a chylothorax.

**Case presentation:**

A 62-year-old Dutch man with a medical history of gastric cancer, treated with neoadjuvant chemotherapy and surgery 13 months prior, presented with dyspnea and a swollen left arm. Computed tomography thorax showed bilateral pleural effusion that was more prominent on the left side. The computed tomography scan further revealed thrombosis of the left jugular and subclavian veins and osseal masses suggesting cancer metastasis. Thoracentesis was performed to confirm the suspicion of gastric cancer metastasis. The obtained fluid was milky with a high level of triglycerides, but contained no malignant cells; hence, the diagnosis of the pleural effusion was chylothorax. Treatment with anticoagulation and a medium-chain-triglycerides diet was started. Furthermore, bone metastasis was confirmed with a bone biopsy.

**Conclusion:**

Our case report demonstrates chylothorax as a rare cause of dyspnea in a patient with pleural effusion and a history of cancer. Therefore, this diagnosis should be considered in all patients with a history of cancer with new-onset pleural effusion and thrombosis of the upper extremities or clavicular/mediastinal lymphadenopathy.

## Background

Chylothorax is a rare condition characterized by leakage of chyle in the thoracic cavity. Lymph fluid or chyle is produced in the intestinal wall, and mainly consists of long-chain triglycerides derived from nutrients in the form of chylomicrons. Chyle consists of T-lymphocytes, electrolytes, fat-soluble vitamins, and proteins. Depending on the amount of fat in the diet, the transport of lymph fluid can differ between 1.5 and 2.5 L a day. Lymph fluid flows through the cisterna chyli and the thoracic duct, and ultimately empties in the venous system at the junction of the left internal jugular and subclavian veins [1–3]. The left upper lung lobe drains lymph fluid to the thoracic duct. The drainage of lymph fluid of the right lung and the left lower lobe is predominantly through the bronchomediastinal trunk to the right subclavian vein [2, 3].

There are various causes of chylothorax. The most common cause is dysfunction of the thoracic duct or its suppliers, which could be caused by obstruction (for example, lymphomas) or trauma (for example, surgery). Other causes are nonsurgical trauma (for example, whiplash and seizures), malignancies (other than lymphoma), lupus, tuberculosis, sarcoidosis, and amyloidosis [1, 2].

A rare cause of chylothorax is (bilateral) thrombosis of the upper extremities or central venous thrombosis, occasionally provoked by central venous catheters [4–22].

The signs and symptoms of a chylothorax differ depending on the amount of chyle leakage and the underlying cause. If a large amount of chyle leaks in the pleural space, immunological weakness, hypoalbuminemia, and lymphopenia can occur. Diagnosis is confirmed with thoracentesis showing milky fluid, triglycerides > 1.24 mmol/L, or triglycerides between 0.56 and 1.24 mmol/L, with chylomicrons present in the pleural fluid. The treatment of chylothorax includes drainage and a low-fat diet with medium-chain triglycerides (MCT diet), which means exclusion of long-chain triglycerides (LCTs), preventing malnutrition, and treating the underlying cause [23].

Diagnosing chylothorax, by assessing the aspect of the pleural fluid, measuring triglycerides and chylomicrons in pleural effusion, and timely starting of treatment, can help respiratory distress, immunosuppression, and malnutrition.

## Case presentation

A 62-year-old Dutch male was diagnosed with a T3N2M0 diffuse type gastric cancer in 2017. His initial symptoms were weight loss and dysphagia. Imaging and gastroscopy showed an ulcer in the gastric antrum and positron emission tomography–computed tomography (PET–CT) showed negative lymph nodes in the omentum. The histological diagnosis was diffuse type adenocarcinoma. The patient was treated in a curative setting with neoadjuvant chemotherapy (epirubicin, oxaliplatin, and capecitabine) followed by laparoscopic gastrectomy. Postsurgery staging showed ypT2N3M0R0. Adjuvant chemotherapy was not administered after very careful consideration because of persistent nausea and poor intake postsurgery including antiemetics, antidiarrheals, and additional nutrition. He did not have the right physical condition to endure adjuvant chemotherapy and its possible side effects. Therefore, the oncologist decided not to pursue adjuvant chemotherapy.

During follow-up at 11 months, a CT-thorax-abdomen was performed due to weight loss. The CT showed multiple mesenterial (maximum 11 mm), multiple retroperitoneal (maximum 14 mm), a few mediastinal (maximum 11 mm), and one left retroclavicular (11 mm) lymphadenopathy. No mass was identifiable as recurrence in the gastric region and no distant metastasis were seen. None of the masses were technically suitable for biopsy (to obtain histology). The recurrence of cancer could therefore not be established but was still suspected, and follow-up was planned after 2 months.

After 2 months, the patient slowly developed abdominal pain, edema of the legs, and dyspnea. CT scan showed pleural effusion but insufficient amount for thoracentesis.

**Fig. 1:**
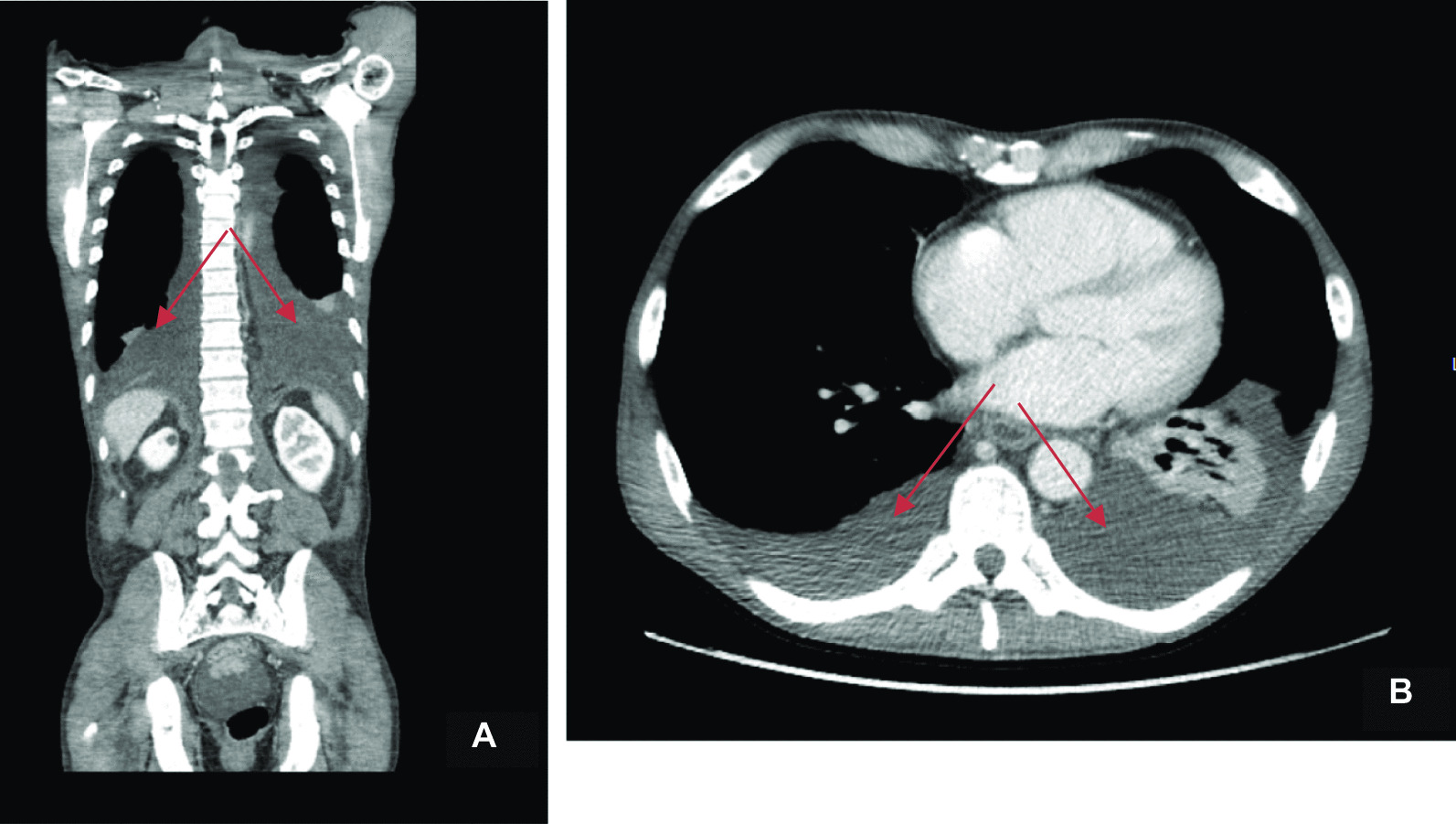
**A**, **B** CT chest–abdomen showing bilateral pleural effusion (indicated by arrows)

**Fig. 2:**
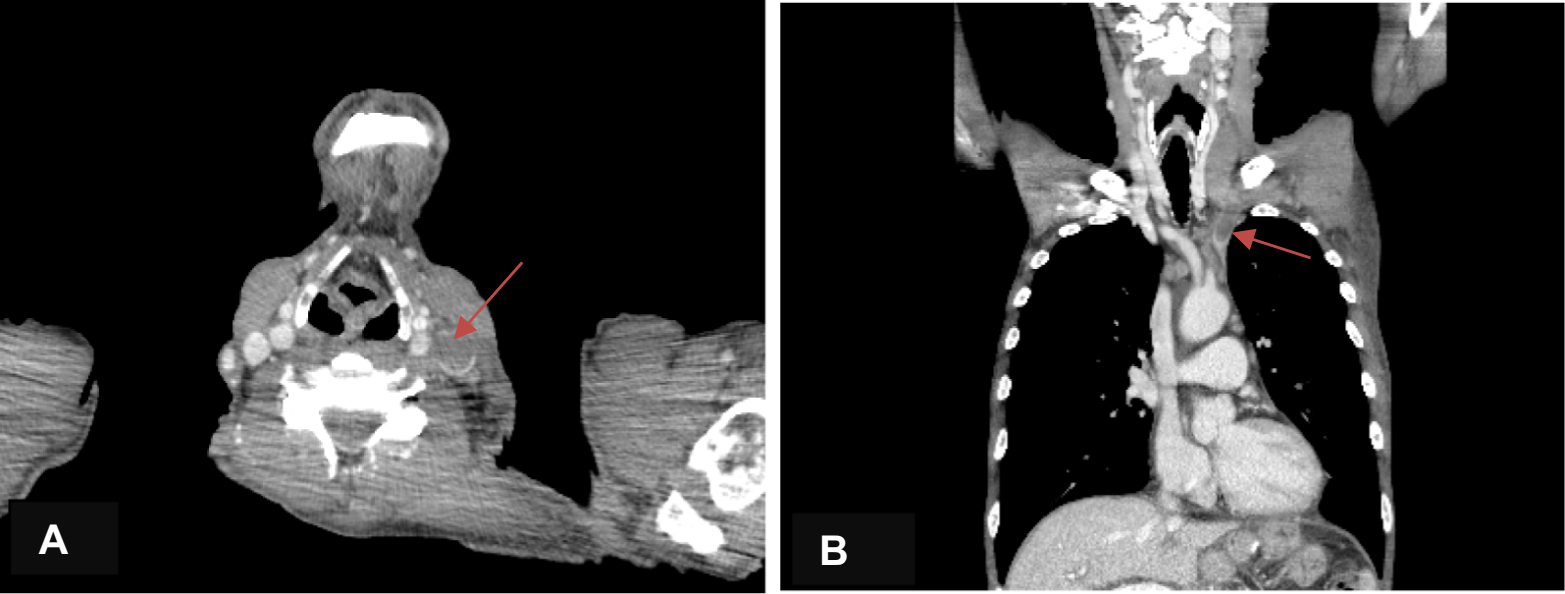
**A**, **B** Thrombosis in the left internal jugular vein and Thrombosis in the left brachiocephalic-subclavian vein (indicated by arrows)

**Fig. 3:**
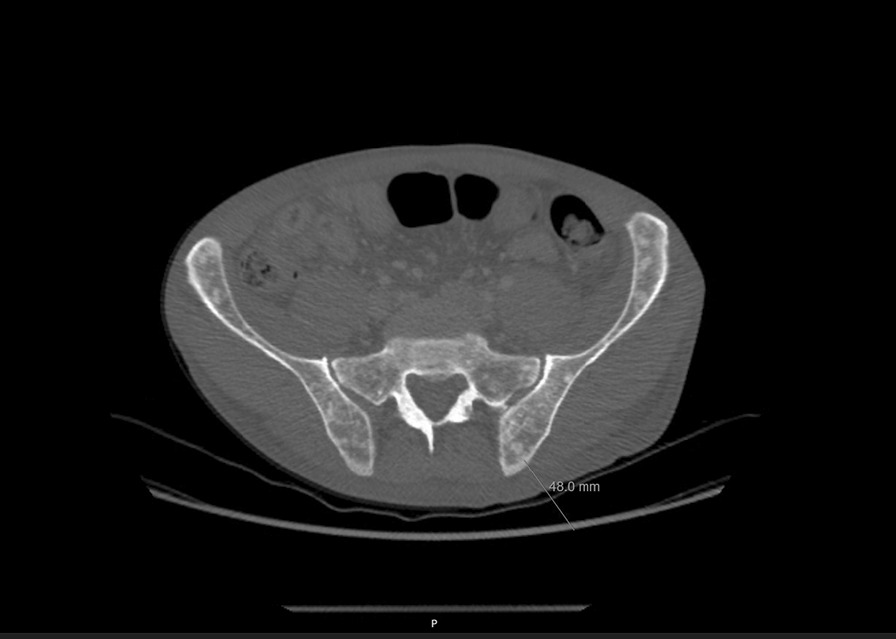
Multiple bone metastasis in spine and pelvis

A few months later the patient developed progressive weakening and edema of the left arm with shoulder pain. Table 1 presents the laboratory findings. Another CT scan was performed showing bilateral pleural effusion (Fig. 1A, B), with more pronounced pleural effusion on the left side, thrombosis of the jugular vein up to the subclavian vein (see Fig. 2A, B), and diffuse osseal masses in the vertebral column and pelvis (see Fig. 3). Pleuritic carcinomatosis was suspected, and therefore thoracentesis was planned before starting anticoagulation.

**Table 1:** Laboratory assessments

		Reference range
White blood cell (/nl)	12.7	4.0–10.0
Hemoglobin (mmol/L)	9.6	8.5–11.0
Platelet (/nl)	184	150–400/nl
Neutrophil (/nl)	10.8	1.5–7.5
Sodium (mmol/L)	137	135–145
Chlorine (mmol/L)	103	97–107
Potassium (mmol/L)	4.1	3.5–5.0
Creatinine (μmol/L)	89	
eGFR (mL/minute)	79	> 90
Blood urea nitrogen (mmol/L)	4.5	2.5–7.5
Calcium (mmol/L)	2.17	2.2–2.65
Phosphate (mmol/L)	0.84	0.9–1.5
Albumin (g/L)	23.4	35–55
CRP (mg/L)	8.8	< 10
Total bilirubin (μmol/L)	4	< 17
Alkaline phosphatase (U/L)	535	35–120
γ‐glutamyltransferase (U/L)	17	< 50
Aspartate aminotransferase (U/L)	19	< 35
Alanine aminotransferase (U/L)	6	< 45
Glucose (mmol/L)	6.6	< 11.1

*eGFR* estimated glomerular filtration rate, *CRP* C‐reactive protein

An uncomplicated thoracentesis was conducted on the left side, removing 20 mL milky fluid. Analysis of the fluid revealed elevated levels of triglycerides (3.3 mmol/L) without malignant cells (Table 2). The milky/yellow aspect of the fluid, in combination with the increased level of triglycerides, confirmed the diagnosis of a chylothorax. Anticoagulation was started along with a MCT diet. A biopsy of one of the osseal masses was planned.

**Table 2:** Laboratory data—pleural fluid examination

Pleural fluid	
Total protein (g/L)	27
LDH (U/L)	87
Cholesterol (mmol/L)	1.7
Triglyceride (mmol/L)	3.3
Glucose (mmol/L)	6.0
Albumin (g/L)	16.8
pH	7.76

*LDH* lactate dehydrogenase

Histology of one osseal mass confirmed the recurrence of gastric cancer, showing a poorly differentiated carcinoma. After considering the possible benefits and side effects of palliative chemotherapy, and taking the clinical condition of the patient into consideration, chemotherapy was not considered feasible and the patient was referred back to the general practitioner with best supportive care. The patient passed away a few months later.

## Discussion

In the current case report we illustrate a rare cause of pleural effusion. We recommend that chyle leakage should be considered in patients with gastric cancer, thrombosis of the upper extremities, and new-onset pleural effusion. The thoracentesis led to the diagnosis of chyle leakage in the thorax caused by thrombosis of the subclavian and jugular veins in a prothrombotic state of a patient with metastatic cancer. The thrombosis of the subclavian and jugular veins subsequently led to a dysfunctional thoracic duct, due to obstruction and high pressure in the thoracic duct, leading to the formation of collateral ducts. The thoracic duct is able to resist high pressure and is not easily ruptured. However, high pressure in the collaterals can lead to backflow of chyle into the lymphatic ducts of the parietal pleura, which subsequently causes chyle leakage in the thoracic cavity through fissures and ruptures.

Theoretically, our patient should have developed isolated left-sided pleural effusion caused by chyle leakage in the left lung as the drainage system of the right lung mostly does not involve the thoracic duct and the subclavian and jugular veins. However, other case reports have also described bilateral chylothorax with one-sided thrombosis in the upper extremities without thrombosis in the lower part of the central venous system (for example vena cava inferior) [7, 10, 13, 17]. Another possible explanations of the right-sided pleural effusion in our patient could be the rapid decrease in albumin. In addition, our patient suffered from edema in the extremities (that is, scrotal and legs), without any other explanation besides the hypoalbuminemia. Hypoalbuminemia is also one of the complications of a chylothorax (leakage of proteins).

In patients undergoing chemotherapy, central venous catheters may cause thrombosis in the upper extremities, or may lead to direct trauma to the thoracic duct or nearby structures, which can lead to a chylothorax [1, 6, 15, 20, 24]. Our patient had a peripherally inserted central catheter (PICC) on the right arm during treatment with chemotherapy in 2017. The chylothorax occurred 1 year after the placement of the PICC. Moreover, the PICC was placed at the right arm whereas the thrombosis occurred in the left subclavian vein and jugular vein, making it unlikely that the thrombosis is a direct result of the peripherally inserted central catheter placement.

Lymphomas can cause direct trauma to the thoracic duct and chylous leakage. In our case, only one retroclavicular lymph node and a few small mediastinal ones (all < 11 mm) were seen. The extent of the thrombosis in contrast to a few small lymph nodes makes the thrombosis certainly more likely to be the cause the chylous pleural effusion in our case.

With increasing indications for PICC, other central venous catheters, and prolonged treatments of cancer patients, more direct trauma and thrombosis in the upper extremities could be expected, which could potentially lead to chylothorax. Some other risk factors for thrombosis in the upper extremities are oral anticonception, nephrotic syndrome, and coagulation disorders. Multiple case reports have already described thrombosis of the upper extremities as a cause of chylothorax. This group of patients is diverse, describing children, infants, catheter-associated thrombosis, spontaneous one-sided chylothorax, and bilateral chylothorax [4–22].

Gastric cancer is described to be associated with chylothorax on its own [25–32]. Devaraj *et al*. [25] described a case report and reviewed six other patients with chylothorax with initial presentation of gastric cancer, or developing chylothorax as a complication of therapy. In addition, three other case reports also described chylothorax due to upper extremity thrombosis in patients with gastric cancer [8, 16, 17]. The etiology of the chylothorax is not always clear. However, in most patients, as in our patient, it is often a late manifestation of occult gastric adenocarcinoma, followed by a fulminant course and death within months after diagnosis.

## Conclusion

Thrombosis of the upper extremities is a rare cause of chylothorax, in which leakage of chyle in the thoracic cavity can potentially lead to severe complications. In most cancer patients, dyspnea is caused by the primary tumor, pulmonary metastasis, lung embolism, or pleural effusion caused by pleuritis carcinomatosa. Our case demonstrates another rare cause of dyspnea with pleural effusion in a patient with cancer. We recommend that chylothorax should be considered as a cause for dyspnea when pleural effusion and thrombosis of the upper extremities are present.

## Data Availability

Not applicable.
